# Retraction: The Effect of Topical Benzydamine Hydrochloride and Cuff Pressure Monitorization on Postoperative Sore Throat Due to Intubation

**DOI:** 10.7759/cureus.r216

**Published:** 2026-01-22

**Authors:** Bilge Olgun Keleş, Menşure Kaya

**Affiliations:** 1 Department of Anesthesiology and Reanimation, Giresun University, Giresun, TUR; 2 Department of Anesthesiology and Reanimation, University of Health Science, Dr. Abdurrahman Yurtaslan Oncology Training and Research Hospital, Ankara, TUR

This article has been retracted by the Editor-in-Chief due to fundamental failures in trial governance and data integrity. The published results include multiple mathematically impossible percentages with missing raw counts despite being reported as n (%), and variables described as collected in the Methods sections are not reported elsewhere. Subsequent corrections submitted by the authors were found to contain additional errors, rendering the findings unreliable. The authors disagree with the decision to retract.

